# Female reproduction and viral infection in a long‐lived mammal

**DOI:** 10.1111/1365-2656.13799

**Published:** 2022-08-21

**Authors:** Jacob D. Negrey, Melissa Emery Thompson, Christopher D. Dunn, Emily Otali, Richard W. Wrangham, John C. Mitani, Zarin P. Machanda, Martin N. Muller, Kevin E. Langergraber, Tony L. Goldberg

**Affiliations:** ^1^ Department of Pathobiological Sciences University of Wisconsin‐Madison Madison WI USA; ^2^ Department of Anthropology University of New Mexico Albuquerque NM USA; ^3^ Makerere University Kampala Uganda; ^4^ Department of Human Evolutionary Biology Harvard University Cambridge MA USA; ^5^ Department of Anthropology University of Michigan Ann Arbor MI USA; ^6^ Department of Anthropology Tufts University Medford MA USA; ^7^ School of Human Evolution and Social Change Arizona State University Tempe AZ USA; ^8^ Institute of Human Origins Arizona State University Tempe AZ USA

**Keywords:** chimpanzee, lactation, life history, metagenomics, pregnancy, primates, reproduction, virus

## Abstract

1. For energetically limited organisms, life‐history theory predicts trade‐offs between reproductive effort and somatic maintenance. This is especially true of female mammals, for whom reproduction presents multifarious energetic and physiological demands.
2. Here, we examine longitudinal changes in the gut virome (viral community) with respect to reproductive status in wild mature female chimpanzees *Pan troglodytes schweinfurthii* from two communities, Kanyawara and Ngogo, in Kibale National Park, Uganda.
3. We used metagenomic methods to characterize viromes of individual chimpanzees while they were cycling, pregnant and lactating.
4. Females from Kanyawara, whose territory abuts the park's boundary, had higher viral richness and loads (relative quantity of viral sequences) than females from Ngogo, whose territory is more energetically rich and located farther from large human settlements. Viral richness (total number of distinct viruses per sample) was higher when females were lactating than when cycling or pregnant. In pregnant females, viral richness increased with estimated day of gestation. Richness did not vary with age, in contrast to prior research showing increased viral abundance in older males from these same communities.
5. Our results provide evidence of short‐term physiological trade‐offs between reproduction and infection, which are often hypothesized to constrain health in long‐lived species.

## Graphical abstract

Trade‐offs between reproductive effort and somatic maintenance (e.g. immune investment) are difficult to study in long‐lived mammals, especially when noninvasive methods are required. By applying metagenomic methods to excreta, researchers investigated such trade‐offs in free‐ranging female chimpanzees. Researchers found increased viral burdens during late pregnancy and lactation, indicating short‐term trade‐offs.

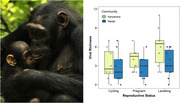

## 1 INTRODUCTION

Life‐history theory predicts that organisms make trade‐offs between reproductive effort and somatic maintenance when energy is limited (Zuk & Stoehr, 2002). These trade‐offs are expected to be particularly pronounced in female mammals, for whom reproduction requires large and prolonged energetic investments. Although gestation is energetically costly, especially during the third trimester (Butte et al., 2004), lactation is more costly still (Clutton‐Brock et al., 1989; Emery Thompson, 2013; Hanwell & Peaker, 1977). Early lactation, when milk constitutes the sole contribution to offspring diet, can even result in negative maternal energy balance (Alam et al., 2003). Given the amount of energy required, lactation can affect other energetically costly processes, including maintenance of physical condition [e.g. bone density (Zeni et al., 1999) and immunity (Ross et al., 1993)]. In humans, frequency and duration of breastfeeding are positively correlated with rate of postpartum weight loss (Dewey et al., 1993), a pattern driven by related physiological changes [e.g. reversal of insulin resistance (Stuebe & Rich‐Edwards, 2009)].

Reproduction also imposes immunological challenges that do not result from energetic trade‐offs. Notably, pregnancy requires an immune ‘balancing act’ to promote fertilization and embryonic implantation, prevent foetal loss, and ultimately induce parturition (Aghaeepour et al., 2017; Clark & Schust, 2013; Gomez‐Lopez et al., 2014; Raghupathy, 1997). Consequently, the maternal immune system undergoes considerable remodelling pre‐, peri‐ and postimplantation. Although these changes are numerous and interactive, a notable pattern in humans is that inflammatory immunity is generally downregulated in favour of humoral and anti‐inflammatory immunity (Raghupathy, 1997; Wang, Sung, et al., 2020). These changes may increase susceptibility to and severity of intracellular infections (Sappenfield et al., 2013). At the same time, females may exhibit prominent behavioural shifts during pregnancy—for example, decreased gregariousness in chimpanzees *Pan troglodytes* (Pusey et al., 2008)—which in turn may modulate exposure to socially transmitted infections.

Reproductive investment and concomitant trade‐offs may have long‐term health consequences. Given scarce resources, an organism's reproductive success often benefits from prioritizing reproductive investment over cellular repair, leading to accumulated cellular damage (Kirkwood, 1977; Kirkwood & Austad, 2000). Thus, as posited by the disposable soma theory, short‐term physiological challenges may contribute to cumulative cellular insult and ageing (Kirkwood, 1977; Kirkwood & Austad, 2000). Although the exact mechanisms by which pathogens cause cumulative somatic effects are not fully understood (McHugh & Gil, 2018), infections contribute to processes such as oxidative stress (Beck et al., 2000; Butcher et al., 2017; Gong et al., 2001) that in turn contribute to ageing (Liguori et al., 2018). Subsequent immunosenescence, or ageing of the immune system, is reflected in turn by increased susceptibility to and severity of infectious disease with age (Krone et al., 2014; Leng & Goldstein, 2010; Thomasini et al., 2017).

Long‐lived nonhuman species are useful models for understanding trade‐offs between reproduction and somatic maintenance. For instance, in chacma baboons *Papio ursinus*, helminth intensity increases during both pregnancy and lactation (Habig et al., 2021), and in red deer *Cervus elaphus*, lactating females have higher parasite counts than females in other reproductive states (Albery et al., 2021). In African buffalo *Syncerus caffer*, anthelmintic treatment increased body condition, which in turn predicted reproductive output (Budischak et al., 2018). However, direct measurements of declining immune function with age are rare in free‐ranging animals (Peters et al., 2019). Notable examples include increased inflammation in ageing Soay sheep *Ovis aries* (Nussey et al., 2012) and age‐related increases in both gastrointestinal parasites and proinflammatory biomarkers in roe deer *Capreolus capreolus* (Cheynel et al., 2017).

Chimpanzees *P. troglodytes* and bonobos *P. paniscus*, our closest living relatives, are continuous (i.e. nonseasonal) breeders (Heldstab et al., 2021) and can live for more than 60 years in their natural habitats (Muller & Wrangham, 2014; Wood et al., 2017). Prior studies of wild female chimpanzees provide equivocal evidence for physiological trade‐offs associated with reproduction. In one study in Kibale National Park, Uganda, chimpanzees shed greater quantities of *Oesophagostomum* during pregnancy and early lactation, with highest shedding several months postpartum and a prominent decrease thereafter (Phillips et al., 2020). In other studies, females were more likely to test positive for *Plasmodium* during late pregnancy than during other stages of reproduction (De Nys et al., 2014), and pregnant but not lactating females exhibited higher levels of neopterin, a proinflammatory biomarker (Negrey et al., 2021). However, long‐term data also suggest that reproductive status does not predict the occurrence of clinically observable respiratory disease (Emery Thompson et al., 2018). Also, some but not all chimpanzees (González et al., 2020) exhibit higher levels of neopterin with age (Negrey et al., 2021), and high‐fertility females in some populations experience less of an age‐related increase in parasite loads than low‐fertility females (Phillips et al., 2020). Finally, in contrast with helminthic parasites, *Plasmodium* detection rates decrease with age, presumably due to acquired immunity (De Nys et al., 2014).

Viruses may be particularly informative for understanding reproductive trade‐offs and immunosenescence in long‐lived mammals because viruses are obligate molecular parasites and thus replicate only through direct co‐option of host intracellular machinery (Rivers, 1927; Ryu, 2017). In prior studies, we found that viral shedding in faeces increased with age in male but not in female chimpanzees in Kibale (Negrey et al., 2020, 2022), and that both males and females shed more viruses when exhibiting clinical signs of illness than when apparently healthy (Negrey et al., 2022). In the present study, we assess changes in the gastrointestinal virome as female chimpanzees transition among reproductive states (cycling, pregnant, lactating). Specifically, we tested whether the energetically costly states of pregnancy and lactation affect the diversity (richness) and quantity (load) of viruses shed in the faeces of mature female chimpanzees. We also assessed whether gastrointestinal viral richness and load increased in female chimpanzees with age, mirroring patterns observed for males in this population (Negrey et al., 2020, 2022).

## 2 MATERIALS AND METHODS

### 2.1 Ethics statement

The Uganda Wildlife Authority and Uganda National Council for Science and Technology approved our noninvasive research protocols. Further approvals were provided by the Institutional Animal Care and Use Committees (IACUCs) of Harvard University (protocol 96‐03), Tufts University (protocol M2019‐83), University of Wisconsin‐Madison (protocol V005039‐R02), and the University of New Mexico (protocol 18‐200,739‐MC). The University of Michigan's IACUC formally exempted our protocols from review.

### 2.2 Study subjects and sample collection

We collected observational data and faecal samples from the Kanyawara and Ngogo chimpanzee communities of Kibale National Park, Uganda, from July 2015 to June 2019. The Kanyawara and Ngogo communities, separated by approximately 10 km (Figure S1), have been the subjects of continuous study since 1987 and 1995 respectively (Muller & Wrangham, 2014; Wood et al., 2017). Early in the study period (1 January 2016), Kanyawara and Ngogo comprised 49 and 194 individuals, more than a third of whom were sexually mature females (i.e. those with known offspring and/or full sexual swellings when cycling). As previously described (Negrey et al., 2020, 2022), we collected faecal samples from individually identified chimpanzees on a quarterly basis and preserved them at a 1:1 ratio with RNA later buffer (Thermo Fisher Scientific). We stored samples in the field in freezers at −20°C until transporting them on ice to the United States for analysis.

### 2.3 Viral identification and quantification

We analysed 60 samples from 16 reproductively mature female chimpanzees (Kanyawara = 8; Ngogo = 8) ranging in age from 11 to 50 years. For each individual, we analysed at least one sample collected prior to pregnancy, one sample collected during pregnancy and one sample collected during early lactation (within a year of parturition, which encompasses peak nursing effort [Bădescu et al., 2017; Emery Thompson et al., 2012]). We identified and quantified viruses in chimpanzee faeces using previously described methods (Goldberg et al., 2017, 2018, 2019; Negrey et al., 2020; Sibley et al., 2016; Toohey‐Kurth et al., 2017). Briefly, we extracted viral nucleic acids from faecal samples, which we then sequenced on an Illumina MiSeq instrument (Illumina) and reconstructed using CLC Genomics Workbench (CLC bio) (see Supporting Information). For analysis of relationships between reproductive status, age and viruses, we only retained viral sequences with mammalian hosts; we discarded viral sequences of bacteriophages as well as viruses likely associated with dietary items (e.g. viruses of figs). We calculated a metagenomic measure of viral load for each sample as the proportion of reads mapping to a viral sequence, normalized to 1 million reads and adjusted for the target sequence length. The resulting metric, viral reads per million per kilobase of target (vRPM/kb), has been validated by real‐time quantitative polymerase chain reaction (Huang et al., 2019; Toohey‐Kurth et al., 2017) and has previously been used to successfully quantify viral loads in wild chimpanzees (Negrey et al., 2020, 2022).

### 2.4 Inferential statistics

We calculated viral prevalence by reproductive status and study community using the modified Wald method (Agresti & Coull, 1998). We then used linear mixed models (LMMs) in R v4.0.3 (R Core Team, 2020) to analyse variation in viral richness (the number of viral species per sample) and total viral load (vRPM/kb of all viruses) by reproductive status, age and study community. Data were approximately normally distributed; we therefore fitted LMMs with Gaussian error structures and restricted maximum likelihood using the ‘lmer’ function in package lmerTest v3.1.3 (Kuznetsova et al., 2017). Because we observed mild heteroscedasticity in model residuals, we ran both models again as robust linear mixed models (RLMMs). The LMM and RLMM results did not differ appreciably, so we therefore report LMM results in the text. We report full LMM and RLMM results in the Supporting Information (Tables S2 and S3).

To determine the relative contributions of each virus to patterns detected in LMMs, we generated random forests using the ‘randomForest’ function in R package randomForest (Liaw & Wiener, 2002), as previously described (Negrey et al., 2022). The random forest algorithm is a machine learning tool that predicts or classifies values by generating a series of decision trees (Breiman, 2001). We created random forests to assess contributions of individual viruses to patterns detected by mixed modelling, with respect to viral richness by reproductive status, richness by community and total viral load by community (see Supporting Information).

## 3 RESULTS

We detected 27 viruses in 60 faecal samples from 16 female chimpanzees (Table 1), including 15 viruses previously identified and reported in this population (Negrey et al., 2020) and 12 novel viruses (Figure S2). Amino acid similarity to known viruses ranged from 46.31% to 99.8% (Table 1). Overall prevalence varied from 1.7% (astrovirus, enterovirus A and unclassified ssDNA virus 3) to 50% (circovirus 1) (Table S1). Of these, 19 viruses were present in both the Kanyawara and Ngogo communities. Enterovirus A, genomovirus and three unclassified ssDNA viruses were identified only in samples from Kanyawara. An astrovirus, chimpanzee stool‐associated RNA virus (chisavirus) and unclassified ssDNA virus 3 were identified only in samples from Ngogo. Viruses that occurred only at Kanyawara appeared in as many as 26.7% of samples from the community (ssDNA virus 5), whereas viruses observed only at Ngogo were present at low prevalence, appearing in no more than 6.7% of samples from the community (ssDNA virus 3). We found an absence of viruses in 4 of the 60 samples.

**TABLE 1:** Viruses detected in 60 faecal samples from 16 female chimpanzees in Kibale National Park, Uganda

	Virus	Closest match (accession number^a^)	Family	Host (country, year)	Genome	Length (nt)^b^	% Identity^c^	Accession number^d^
1	Chimpanzee adenovirus	chimpanzee adenovirus Y25 (YP_006272954)	*Adenoviridae*	Chimpanzee (−, 1969)	dsDNA	2403	99.75	MW876510
2	Chimpanzee astrovirus	human astrovirus BF34 (YP_009047078)	*Astroviridae*	Human (Burkina Faso, 2010)	ssRNA	1044	79.25	ON304043
3	Chimpanzee bufavirus (protoparvovirus)	protoparvovirus (QFX66146)	*Parvoviridae*	Macaque (Thailand, 2017)	ssDNA	880	65.75	ON304047
4	Chimpanzee circovirus 1	dromedary stool‐associated circular ssDNA virus (AIY31253)	*Circoviridae*	Camel (United Arab Emirates, 2013)	ssDNA	1134	60.65	MW876514
5	Chimpanzee circovirus 2	circovirus sp. (QBA83760)	*Circoviridae*	Pig (China, 2017)	ssDNA	1179	46.31	MW876515
6	Chimpanzee enterovirus A	enterovirus A76 (AXG24379)	*Picornaviridae*	Human (India, 2010)	ssRNA	5509	96.89	ON304044
7	Chimpanzee enterovirus D	enterovirus D94 (QRF54819)	*Picornaviridae*	Human (Niger, 2014)	ssRNA	6573	96.85	ON304045
8	Chimpanzee genomovirus	*Genomoviridae* sp. (QCW23657)	*Genomoviridae*	Mouse (USA, 2017)	ssDNA	712	92.23	ON304048
9	Chimpanzee kobuvirus	kobuvirus sp. (AVH76469)	*Picornaviridae*	Human (Vietnam, 2014)	ssRNA	782	99.62	ON304046
10	Chimpanzee picobirna‐like virus	kumba picobirna‐like virus (QAA77647)	Unclassified Picornavirales	Human (Cameroon, 2014)	RNA	1188	93.43	MT076202
11	Chimpanzee salivirus	salivirus FHB (YP_009067077)	*Picornaviridae*	Human (China, 2011)	ssRNA	7125	98.27	MW876512
12	Chisavirus (chimpanzee stool‐associated RNA virus)	husavirus (AWU65954)	Unclassified Picornavirales	Human (Venezuela, 2015)	ssRNA	8379	62.95	MW876513
13	Eastern chimpanzee associated porprismacovirus 1	macaca mulatta faeces associated virus 4 (APG55823)	*Smacoviridae*	Rhesus macaque (USA, 2014)	ssDNA	777	64.00	MT076205
14	Eastern chimpanzee associated porprismacovirus 2	porprismacovirus sp. (QRV61735)	*Smacoviridae*	Pig (China, 2017)	ssDNA	735	73.62	MT076206
15	Eastern chimpanzee associated porprismacovirus 3	chicken smacovirus mg7_67 (QIR82279)	*Smacoviridae*	Chicken (USA, 2017)	ssDNA	786	55.47	MT076207
16	Eastern chimpanzee associated porprismacovirus 4	chimpanzee stool‐associated circular ssDNA virus (ADB24816)	*Smacoviridae*	Chimpanzee (Tanzania, 2004)	ssDNA	816	99.26	MT076208
17	Eastern chimpanzee associated porprismacovirus 6	porprismacovirus sp. (QRV61732)	*Smacoviridae*	Pig (China, 2017)	ssDNA	780	68.73	MT076210
18	Eastern chimpanzee associated porprismacovirus 7	chimpanzee associated porprismacovirus 2 (YP_009508863)	*Smacoviridae*	Chimpanzee (Tanzania, 2004)	ssDNA	586	92.27	MW876516
19	Eastern chimpanzee associated porprismacovirus 8	chicken smacovirus mg4_964 (QIR82267)	*Smacoviridae*	Chicken (USA, 2017)	ssDNA	786	77.27	MW876517
20	Eastern chimpanzee associated porprismacovirus 9	chicken smacovirus mg4_964 (QIR82267)	*Smacoviridae*	Chicken (USA, 2017)	ssDNA	771	68.98	MW876518
21	Eastern chimpanzee associated porprismacovirus 10	*Smacoviridae* sp. (QIN96629)	*Smacoviridae*	Indian peafowl (China, 2018)	ssDNA	846	73.93	ON304049
22	Eastern chimpanzee associated porprismacovirus 11	eastern chimpanzee associated porprismacovirus 1 (QNH86214)	*Smacoviridae*	Chimpanzee (Uganda, 2016)	ssDNA	535	70.17	ON304050
23	Eastern chimpanzee associated porprismacovirus 12	*Chlorocebus cynosuros* associated smacovirus (BBE29362)	*Smacoviridae*	Malbrouck (Zambia, 2009)	ssDNA	794	52.33	ON304051
24	Unclassified circular ssDNA virus 3	ssDNA virus sp. (QRV62033)	Unclassified	Pig (China, 2017)	ssDNA	861	94.41	ON304052
25	Unclassified circular ssDNA virus 4	circular ssDNA virus sp. (APG55846)	Unclassified	Macaque (USA, 2014)	ssDNA	690	92.00	ON304053
26	Unclassified circular ssDNA virus 5	ssDNA virus sp. (QRV62023)	Unclassified	Pig (China, 2017)	ssDNA	910	58.08	ON304054
27	Unidentified circular ssDNA virus 6	Unidentified circular ssDNA virus (AWU66046)	Unclassified	Human (Venezuela, 2015)	ssDNA	834	94.96	MT076201

^a^ Accession number of closest match in GenBank.

^b^ Length of the nucleotide sequence used to analyse phylogenetic relationships and viral load.

^c^ % amino acid similarity of the new virus to its closest match in GenBank, as determined from query coverage and E‐value.

^d^ Accession number of new viral sequence deposited in GenBank.

**FIGURE 1:**
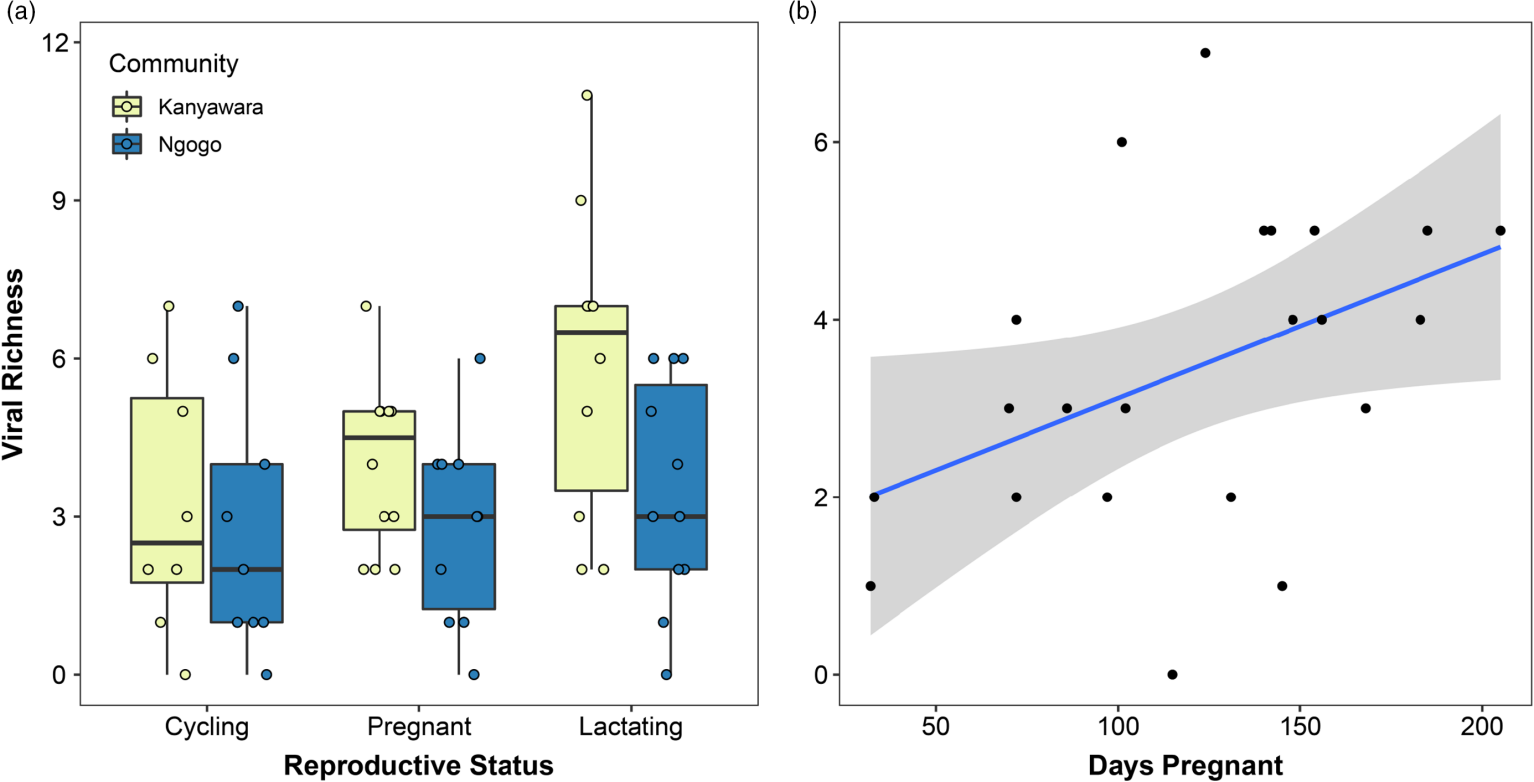
Viral richness as a function of reproductive status in wild female chimpanzees. (a) Richness in cycling, pregnant and lactating chimpanzees. Light and dark boxes represent Kanyawara and Ngogo respectively. The top and bottom of each box represent the 75th and 25th percentiles, respectively, and bold horizontal lines indicate medians. (b) Viral richness by gestation day in pregnant chimpanzees. Shading indicates the 95% confidence interval.

Viral richness increased when individual chimpanzees were lactating compared to when they were cycling (*β* = −1.95, *SE* = 0.720, 95% CI [−3.45, −0.451], *p* = 0.013; Table S2; Figure 1a) or pregnant (*β* = −1.91, *SE* = 0.718, 95% CI [−3.42, −0.415], *p* = 0.015; Table S2; Figure 1a). Mean viral richness among lactating females increased 1.54‐ and 1.34‐fold, respectively, compared to when they were cycling and pregnant (*M* ± *SD*: cycling, 3.00 ± 2.40; pregnant, 3.45 ± 1.77; lactating, 4.62 ± 2.78; Figure 1a). This relationship was not likely due to seasonal effects: a Pearson Chi‐square test did not support an association between collection quarter (e.g. January–March, April–June, etc.) and reproductive status (*X*‐squared = 4.92, *df* = 6, *p* = 0.553). Random forest classification indicated that richness among lactating females was most strongly predicted by unclassified ssDNA virus 5 and porprismacovirus 2 (Table S4). A post‐hoc general additive model indicated a marginally significant increase in viral richness with estimated date of gestation (EDF = 1.00, *F* = 4.70, *p* = 0.042; Figure 1b). The model defaulted to a linear relationship (i.e. did not indicate nonlinearity between richness and date of pregnancy), perhaps due to small sample size of individuals. A post‐hoc mixed model indicated that viral richness did not significantly vary from the second half of pregnancy to lactation (*β* = 0.585, *SE* = 0.829, *p* = 0.488). Among lactating females, richness did not vary by days since parturition (EDF = 1.32, *F* = 1.41, *p* = 0.358). Total viral load did not vary by reproductive status (Table S3) and did not increase with day of gestation (EDF = 1.00, *F* = 0.336, *p* = 0.568).

**FIGURE 2:**
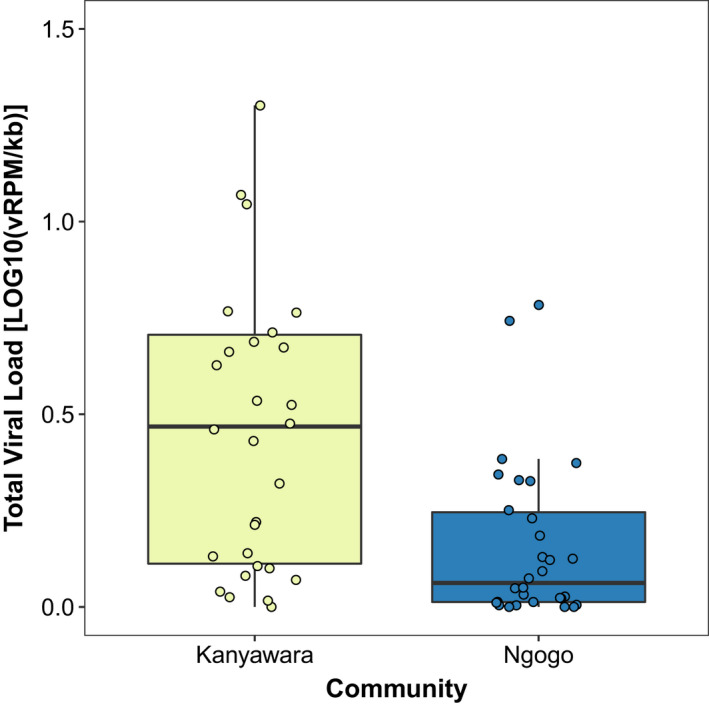
Total viral load (vRPM/kb) of wild female chimpanzees by study community. The upper and lower bounds of each box represent the 75th and 25th percentiles, respectively, and bold horizontal lines indicate medians. Two outliers from the Kanyawara community are above the upper limit of the *y* axis and are not visible.

Viral richness was higher in chimpanzees from Kanyawara than in chimpanzees from Ngogo, regardless of reproductive state (*β* = −1.62, *SE* = 0.720, 95% CI [−2.98, −0.163], *p* = 0.032; Table S2; Figure 1a). Mean viral richness in Kanyawara was approximately 1.5 times higher than in Ngogo (Mean vRPM/kb ± *SD*: Kanyawara, 4.43 ± 2.53; Ngogo, 3.03 ± 2.08). Random forests indicated that unclassified ssDNA virus 5, circovirus 2 and unclassified ssDNA virus 4 most strongly contributed to this effect (Table S4; the two unclassified viruses were found only in samples from Kanyawara). Furthermore, total viral load was lower in samples from Ngogo than in samples from Kanyawara when controlling for female reproductive state (*β* = −0.934, *SE* = 0.281, 95% CI [−1.51, −0.409], *p* = 0.006; Table S3; Figure 2). On average, viral loads were 3.4 times higher at Kanyawara than at Ngogo (*M* ± *SD*: Kanyawara, 0.543 ± 0.540; Ngogo, 0.158 ± 0.208). Random forest classification indicated that circovirus 2 and unclassified ssDNA virus 5 contributed most strongly to this effect (Table S4; Figure S3). We did not include sample collection quarter as a fixed effect in our models to prevent overfitting; however, a Pearson Chi‐square test did not indicate a relationship between collection quarter and study community (*X*‐squared = 2.34, *df* = 3, *p* = 0.505).

Mixed modelling indicated that age was not associated with either viral richness (*β* = 0.033, *SE* = 0.321, 95% CI [−0.672, 0.687], *p* = 0.919; Table S2) or total viral load (*β* = 0.047, *SE* = 0.126, 95% CI [−0.188, 0.289], *p* = 0.717; Table S3).

## 4 DISCUSSION

We used metagenomic methods to identify and quantify viruses shed by wild female chimpanzees over the course of reproduction. We identified 27 viruses, including 12 novel viruses, in 16 female chimpanzees sampled over time in a population in western Uganda. Nineteen viruses (70.4%) were single‐stranded DNA viruses, which are geographically widespread and infect a variety of hosts (Shulman & Davidson, 2017). Consistent with our prior work (Negrey et al., 2020; Negrey et al., 2022), we found that viral shedding varied with host physiology and ecological conditions. In the present study, we observed changes in viral richness and load based on female reproductive state and study community. Viruses that we previously found most strongly associated with illness or old age in males from this same chimpanzee population (e.g. salivirus, chisavirus; Negrey et al., 2020, 2022) did not drive the reproductive patterns observed in females.

Viral richness was higher in females when they were lactating than when they were cycling or pregnant. In addition, viral richness increased as pregnancy progressed. Given the high energetic costs of late pregnancy and early lactation, our findings support the hypothesis that energetic limitations force trade‐offs between reproduction and physiological processes associated with somatic maintenance and immunity. Energetic data from wild chimpanzees in this population have shown that females are particularly energetically stressed in the year following parturition (Emery Thompson et al., 2012), which is when we chose to sample the individuals in this study. Female chimpanzees do not wean their infants until 4–6 years, so we do not yet know how maternal costs change as infants grow and begin consuming solid foods. For example, shedding of *Oesophagostomum* in Kibale chimpanzees peaks in the months following parturition and returns to baseline levels by approximately 2.5 years postpartum (Phillips et al., 2020). The increase in viral richness with progression of pregnancy parallels patterns of *Oesophagostomum* (Phillips et al., 2020) and *Plasmodium* infection (De Nys et al., 2014). Females experience the dual effects of escalating energetic costs and pregnancy‐related shifts in immunoregulation that may each contribute to high viral shedding in the postpartum period. The high costs of lactation appear to compound these effects either by rendering females susceptible to new infections or decreasing their ability to control existing infections.

Kanyawara females exhibited greater viral richness and total viral load than Ngogo females, regardless of reproductive status. This result parallels a previous finding that chimpanzees of any age and sex at Kanyawara exhibited higher viral loads than those at Ngogo when exhibiting clinical signs of ill health (Negrey et al., 2022). The direction of this effect is striking because the Ngogo community is larger than the Kanyawara community, and, other factors being equal, group size is hypothesized to increase pathogen transmission (Freeland, 1976). As previously posited (Negrey et al., 2022), community‐level variation in viral shedding may be attributable to documented differences in dietary quality and energy availability. Specifically, the Ngogo territory contains a greater abundance of ripe fruit (Potts et al., 2011), which corresponds to higher energy balance in Ngogo chimpanzees than in Kanyawara chimpanzees (Emery Thompson et al., 2009). Diet quality impacts immune function (Scrimshaw & SanGiovanni, 1997), so habitat quality may contribute to this trend. In addition, the Kanyawara community territory abuts the park boundary, whereas Ngogo territory does not. Anthropogenic disturbance (e.g. forest fragmentation, poaching) and associated stress (McLennan et al., 2019), exposure to human and domestic animal pathogens directly or through the environment (Goldberg et al., 2008), and exposure to environmental toxins (Wang, Steiniche, et al., 2020) may explain differences between Kanyawara and Ngogo viromes.

Finally, we did not find age‐related variation in either viral richness or load, consistent with our prior research on chimpanzee females (Negrey et al., 2020). The youngest and oldest individuals in the current study (at the time of sample collection) were 11.5 and 50.3 years, a range of 38.8 years. Given this age range, the absence of age‐related variation in the virome is notable, especially because we have observed increases in helminthic parasite shedding with age in females (Phillips et al., 2020) and prominent increases in viral shedding among older males in the same population (Negrey et al., 2020, 2022). These results suggest that patterns of immunosenescence differ between males and females. Life expectancy for female chimpanzees is greater than for males (Muller & Wrangham, 2014; Wood et al., 2017), which may result, in part, from differences in physiological ageing rates—that is, immunosenescence is delayed, slower and/or less pronounced in females than in males, as females likely increase reproductive success through longevity (Rolff, 2002). We note that the virome of the gastrointestinal tract may be less responsive to immunosenescence and/or general physiological health than viromes of other body compartments (e.g. blood, lung), which could have reduced our ability to detect associations between viruses and age in females.

Viruses shed in faeces do not encompass all viruses harboured by an organism. The extent and frequency with which rhinoviruses, for instance, are shed in faeces vary widely among individuals (Savolainen‐Kopra et al., 2013). Given such variability, research seeking to assess the full diversity of viruses harboured by and/or infecting wildlife would ideally examine multiple tissue types and byproducts, from saliva (Smiley Evans et al., 2015) to urine (Newman et al., 1998). We note that Kanyawara and Ngogo chimpanzees do not harbour simian immunodeficiency viruses that occur at high prevalence elsewhere (Santiago Mario et al., 2003), and this difference may influence analyses of infection and life‐history trade‐offs. We also note that viral shedding may ‘spike’ prior to death (either as cause or consequence of declining health), so our data may suffer from survival bias (i.e. individuals who live longer are those able to avoid or fight infection), as has been shown in humans (Vaupel & Yashin, 1985) and wild animals other than chimpanzees (Beirne et al., 2016). Nevertheless, this evidence suggests that the costs of reproductive effort for female immunological health are transient and do not accumulate, as has been observed in some human subsistence groups (Gurven et al., 2016).

We acknowledge limitations in our methods that underscore the difficulty in measuring viral burdens in wild chimpanzees and preclude definitive claims about the eco‐physiological mechanisms underlying our results. Trade‐offs between the energetic demands of pregnancy and lactation and metrics of viral infection may be direct (i.e. reduced robustness or duration of immunological defences against intracellular microbes) or indirect (i.e. resulting secondarily from other factors that covary with reproductive status, even in the absence of immunological effects, e.g. behavioural differences that affect viral exposure). We did not measure immunity or energetic status directly in this study, but that would be a logical next step. Furthermore, many of the viruses we identified remain poorly characterized, and their relationship to host health remains unclear. As we have noted previously (Negrey et al., 2022), several of the identified viruses—notably, salivirus and chisavirus—may represent anthroponoses, and future data from humans living in proximity to Kibale National Park will illuminate such relationships, if they exist. Because our study was observational and noninvasive (essential for research on wild chimpanzees), the viruses we identified could be causes of ill health or benign reflections of physiological compromise. Notably, we did not address changes throughout the menstrual cycle, which in primates has been shown to influence susceptibility to infection [e.g. *Candida albicans* (Steele et al., 1999), simian immunodeficiency virus (Morris et al., 2015)], and influence inflammatory biomarkers (Negrey et al., 2021). Finally, we acknowledge limitations common to viral metagenomics, as indicated by our prior work (Negrey et al., 2022). Although highly specific, viral metagenomics methods can be less sensitive than targeted assays, especially when working with complex biological samples with high background noise and multiple viruses (Chiu & Miller, 2019). Metagenomics approaches are also biased towards viruses with small DNA genomes (Callanan et al., 2021; Roux et al., 2016). In our dataset, for example, 19 of the 27 viruses (70.4%) we report are single‐stranded DNA viruses (Table 1), which may be overrepresented.

Our results may nevertheless be generalizable to other long‐lived mammalian species and have transdisciplinary applications. For instance, understanding the physiological costs of lactation may be applicable to captive and free‐ranging animal management (Moresco & Agnew, 2013). Our results may be particularly pertinent to the reproductive evolution of human females, given similarities in genetics and life histories between humans and chimpanzees. Both species have long life expectancies that vary greatly with local ecological conditions (Wood et al., 2017). Furthermore, both humans and chimpanzees invest great time and energy in offspring (Barton & Capellini, 2011; Martin, 1995). Although studies of humans and nonhuman primates have often highlighted pregnancy as an immunologically costly reproductive stage (e.g. De Nys et al., 2014), our results suggest that hominid females may face greater physiological challenges when lactating. Prior studies have noted the protective effects of breastfeeding on health and mortality in human children (Feachem & Koblinsky, 1984). Our results contribute to this body of knowledge by showing that lactation also affects biomarkers of health in the lactating mother (measures of viral infection in the case of this study). Postnatal care is often neglected relative to prenatal care (Albers, 2000), reflecting the general rarity of prolonged breastfeeding by mothers in industrialized populations and the United States in particular (Dagher et al., 2016). Should our results be generalizable to humans, they suggest that more consideration be given to postnatal infectious disease susceptibility for the benefit of maternal and child health alike.

## AUTHOR CONTRIBUTIONS

Tony L. Goldberg, Melissa Emery Thompson and Jacob D. Negrey conceived the study; Jacob D. Negrey and Tony L. Goldberg drafted the manuscript; Christopher D. Dunn and Jacob D. Negrey performed laboratory work; Jacob D. Negrey completed bioinformatics and statistical analyses; Melissa Emery Thompson, Emily Otali, Richard W. Wrangham, John C. Mitani, Zarin P. Machanda, Martin N. Muller and Kevin E. Langergraber coordinated data collection; all authors made significant intellectual contributions and revised the manuscript.

## CONFLICT OF INTEREST

The authors declare no conflict of interest.

## Supporting information


Appendix S1

## Data Availability

Viral nucleotide sequences have been deposited on GenBank under accession numbers MT076199 to MT076210, MW876510 to MW876520 and ON304043 to ON304054. Data and R code are available on figshare http://doi.org/10.6084/m9.figshare.19653849 (Negrey, 2022).
