# Supplementary material for: Female reproduction and viral infection in a long‐lived mammal
Source: J Anim Ecol. 2022 Aug 21;91(10):1999–2009. doi: 10.1111/1365-2656.13799 (PMC9532343; doi:10.1111/1365-2656.13799)
Supplement: Supplementary file 1 — Appendix S1 [file JANE-91-1999-s001.docx]

**Supporting Information**

**Female reproduction and viral infection in a long-lived mammal**

Jacob D. Negrey, Melissa Emery Thompson, Christopher D. Dunn, Emily Otali, Richard W. Wrangham, John C. Mitani, Zarin P. Machanda, Martin N. Muller, Kevin E. Langergraber, and Tony L. Goldberg

Correspondence to Tony L. Goldberg (tony.goldberg@wisc.edu)

This document contains:

**1. Supplementary methods**

1a. Viromics

1b. Bioinformatics

1c. Inferential Statistics

**2. Supplementary figures**

Figure S1 – Map of Kibale National Park

Figure S2 – Phylogenetic trees of viruses newly identified in Kibale chimpanzees

Figure S3 – Heatmap of viral loads

**3. Supplementary tables**

Table S1 – Viral prevalence by reproductive status and study community

Table S2 – Results of linear mixed models for viral richness

Table S3 – Results of linear mixed models for total viral load

Table S4 – Variable importance values for random forests

**1. Supplementary Methods**

1a. *Viromics*

To identify viruses shed by wild chimpanzees, we employed previously validated metagenomic methods (Sibley *et al.* 2016; Goldberg *et al.* 2017; Toohey-Kurth, Sibley & Goldberg 2017; Goldberg *et al.* 2018; Goldberg *et al.* 2019; Negrey *et al.* 2020; Negrey *et al.* 2022). In brief, we combined 200 µl of feces mixed with RNAlater nucleic acid preservation buffer (Thermo Fisher Scientific, Waltham, Massachusetts, USA) and 800 µl of Hanks’ Balanced Salt Solution. This solution was homogenized by bead beating and then treated with nucleases. We subsequently extracted nucleic acids using the Qiagen QIamp MinElute Virus Spin Kit (Qiagen, Hilden, Germany) and used the extracted RNA to synthesize cDNA. We used Agencourt AmpureXP beads (Beckman Coulter, Brea, CA, USA) to purify cDNA and the Illumina Nextera XT kit (Illumina, San Diego, CA, USA) to prepare libraries. We then pooled and sequenced libraries using 150x150 cycle paired-end chemistry on an Illumina MiSeq instrument.

1b. *Bioinformatics*

We used CLC Genomics Workbench (CLC bio, Aarhus, Denmark) to reconstruct viral sequences. We first trimmed sequences for length and quality, then removed reads mapping to contaminants and chimpanzee DNA. We assembled reads *de novo* and extracted the resulting contiguous sequences (contigs). We used the BLASTn and BLASTx algorithms to compare contigs to those logged in the GenBank database at the nucleotide and amino acid levels (Altschul *et al.* 1990; Gish & States 1993).

We constructed viral phylogenetic trees using polymerase genes whenever possible. We first aligned viral sequences with closely related sequences acquired from GenBank using TranslatorX (Abascal, Zardoya & Telford 2010), using the Gblocks algorithm to remove poorly aligned regions (Castresana 2000). We then generated maximum likelihood phylogenetic trees with 1,000 bootstrap replicates using PhyML v3.0 (Lefort, Longueville & Gascuel 2017) and displayed the final trees using FigTree v1.4.4 (Rambaut 2018).

1c. *Inferential Statistics*

We ran two linear mixed models (LMMs). The response variables were viral richness and total viral load, respectively, and the fixed effects were the chimpanzee’s reproductive status, study community, and age at time of sample collection. We included chimpanzee age, calculated as previously described (Negrey *et al.* 2020), as a continuous variable. We also included the interactions of reproductive status with age and study community. As these interactions resulted in p values greater than alpha (0.05), they were removed from the final models. We also included subject ID as a random intercept and reproductive status by subject ID as a random slope. In the model of viral richness, we additionally included sample collection year and sample collection quarter (e.g., January-March, April-June, etc.) as random intercepts; these variables were excluded from the model for total viral load as they explained little variance and prevented model convergence. We used the Satterthwaite method to estimate degrees of freedom and calculate p values (Luke 2017). We calculated profile 95% confidence intervals using the “confint” function and used the “difflsmeans” function in lmerTest for pairwise comparisons of reproductive status.

We assessed normality and homoskedasticity of model residuals using Shapiro-Wilk tests (Shapiro & Wilk 1965) and by inspecting residual plots, QQ plots, and histograms. Diagnostics indicated mild heteroskedasticity even after transforming data. We therefore ran both models again as robust linear mixed models (RLMMs) using the “rlmer” function in package robustlmm (Koller 2016). Random slopes were excluded from the robust models, as these led to convergence issues. We calculated p values for RLMMs using degrees of freedom derived by Satterthwaite approximations from a non-robust model (Luke 2017), as per Geniole *et al.* (2019). We provide both LMM and RLMM results in Tables S2 and S3.

After implementing mixed models, we generated random forests to assess the relative contributions of individual viruses to observed patterns, as previously described (Negrey *et al.* 2022). In the first forest, we addressed the higher viral richness exhibited by females when lactating than when cycling and pregnant; the response variable was richness in samples from lactating females, and the predictor variables were the prevalence of each virus. In the second and third forests, we addressed community-level variation in viral richness and total viral load. The response variable in both models was study community, and the predictors were viral prevalence and viral load, respectively. Importance values for each virus were calculated as the mean decrease in node impurity (Segal & Xiao 2011) as represented by the Gini index (Liaw & Wiener 2002).

**2. Supplementary Figures**

**Figure S1.** Map of Kibale National Park, Uganda, showing approximate locations of the Kanyawara and Ngogo territories (solid black lines). Shaded area represents forest, and dashed lines represent major public roads.


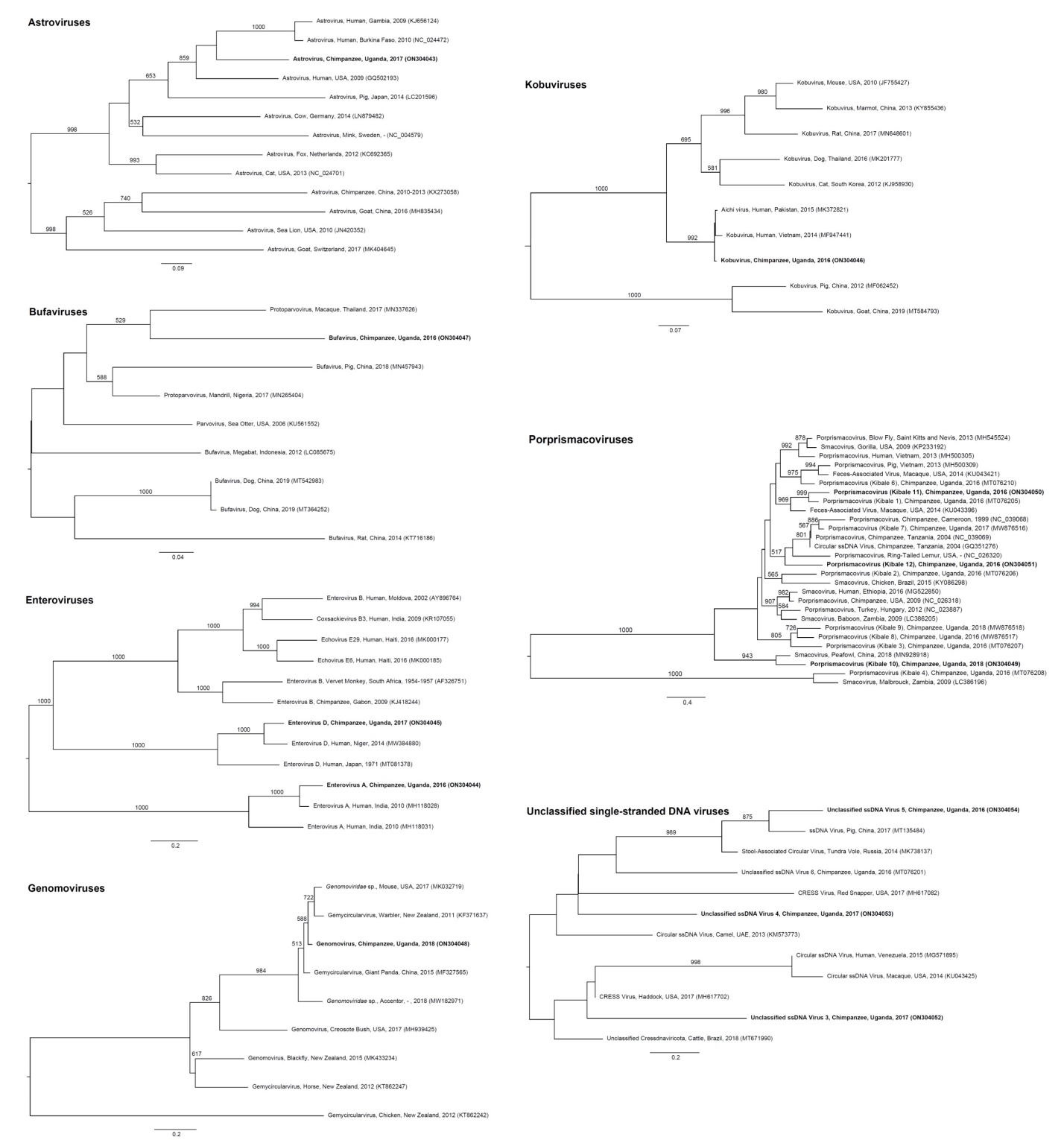


**Figure S2.** Maximum likelihood phylogenetic trees of newly identified viruses (shown in bold) that were isolated from Kanyawara and Ngogo chimpanzee fecal samples. Each label includes the virus name, host organism, country of origin, year of sample collection, and GenBank accession number (in parentheses). Percent confidence resulting from 1,000 bootstrap replicates are shown for all branches with values ≥50%. Nucleotide substitutions per site are indicated by scale bars.

**Figure S3.** Heatmap of viral loads for 60 fecal samples from 16 chimpanzees, organized by study community and reproductive status. Values range from 0 (lightest) to 3.2 Log10(vRPM/kb) (darkest). Each virus has been numbered as per Table 1.

**3. Supplementary Tables**

**Table S1.** Prevalence of 27 viruses detected in fecal samples from 16 female chimpanzees. Upper and lower limits of 95% confidence intervals are listed in parentheses. (n = number of fecal samples)

|  | Virus | Overall (n=60) | Cycling (n=17) | Pregnant (n=22) | Lactating (n=21) | Kanyawara (n=30) | Ngogo (n=30) |
| --- | --- | --- | --- | --- | --- | --- | --- |
| 1 | chimpanzee adenovirus | 20.0 (11.7, 31.9) | 17.7 (5.4, 41.8) | 18.2 (6.7, 39.1) | 23.8 (10.2, 45.5) | 20.0 (9.1, 37.7) | 20.0 (9.1, 37.7) |
| 2 | chimpanzee astrovirus | 1.7 (<0.1, 9.7) | 0.0 (0.0, 21.6) | 4.6 (<0.1, 23.5) | 0.0 (0.0, 18.2) | 0.0 (0.0, 13.5) | 3.3 (<0.1, 18.1) |
| 3 | chimpanzee bufavirus (protoparvovirus) | 10.0 (4.3, 20.5) | 11.8 (2.0, 35.6) | 13.6 (3.9, 34.2) | 4.8 (<0.1, 24.4) | 16.7 (6.9, 34.0) | 3.3 (<0.1, 18.1) |
| 4 | chimpanzee circovirus 1 | 50.0 (37.7, 62.3) | 47.0 (26.2, 69.0) | 40.9 (23.2, 61.3) | 61.9 (40.8, 79.3) | 50.0 (33.2, 66.9) | 50.0 (33.2, 66.9) |
| 5 | chimpanzee circovirus 2 | 25.0 (15.7, 37.3) | 17.7 (5.4, 41.8) | 22.7 (9.7, 43.9) | 33.3 (17.1, 54.8) | 36.7 (21.8, 54.6) | 13.3 (4.7, 30.3) |
| 6 | chimpanzee enterovirus A | 1.7 (<0.1, 9.7) | 5.9 (<0.1, 28.9) | 0.0 (0.0, 17.6) | 0.0 (0.0, 18.2) | 3.3 (<0.1, 18.1) | 0.0 (0.0, 13.5) |
| 7 | chimpanzee enterovirus D | 11.7 (5.5, 22.5) | 11.8 (2.0, 35.6) | 9.1 (1.3, 29.0) | 14.3 (4.1, 35.5) | 16.7 (6.9, 34.0) | 6.7 (0.8, 22.4) |
| 8 | chimpanzee genomovirus | 6.7 (2.2, 16.4) | 0.0 (0.0, 21.6) | 4.6 (<0.1, 23.5) | 14.3 (4.1, 35.5) | 13.3 (4.7, 30.3) | 0.0 (0.0, 13.5) |
| 9 | chimpanzee kobuvirus | 3.3 (0.3, 12.0) | 5.9 (<0.1, 28.9) | 0.0 (0.0, 17.6) | 4.8 (<0.1, 24.4) | 3.3 (<0.1, 18.1) | 3.3 (<0.1, 18.1) |
| 10 | chimpanzee picobirna-like virus | 8.3 (3.2, 18.5) | 0.0 (0.0, 21.6) | 4.6 (<0.1, 23.5) | 19.1 (7.1, 40.6) | 13.3 (4.7, 30.3) | 3.3 (<0.1, 18.1) |
| 11 | chimpanzee salivirus | 20.0 (11.7, 31.9) | 17.7 (5.4, 41.8) | 27.3 (12.9, 48.4) | 14.3 (4.1, 35.5) | 16.7 (6.9, 34.0) | 23.3 (11.5, 41.2) |
| 12 | chisavirus (chimpanzee stool-associated RNA virus) | 3.3 (0.3, 12.0) | 5.9 (<0.1, 28.9) | 4.6 (<0.1, 23.5) | 0.0 (0.0, 18.2) | 0.0 (0.0, 13.5) | 6.7 (0.8, 22.4) |
| 13 | eastern chimpanzee associated porprismacovirus 1 | 31.7 (21.3, 44.3) | 23.5 (9.1, 47.8) | 31.8 (16.2, 52.9) | 38.1 (20.7, 59.2) | 33.3 (19.1, 51.3) | 30.0 (16.5, 48.0) |
| 14 | eastern chimpanzee associated porprismacovirus 2 | 18.3 (10.4, 30.1) | 17.7 (5.4, 41.8) | 9.1 (1.3, 29.0) | 28.6 (13.6, 50.2) | 23.3 (11.5, 41.2) | 13.3 (4.7, 30.3) |
| 15 | eastern chimpanzee associated porprismacovirus 3 | 3.3 (0.3, 12.0) | 0.0 (0.0, 21.6) | 0.0 (0.0, 17.6) | 9.5 (1.5, 30.1) | 3.3 (<0.1, 18.1) | 3.3 (<0.1, 18.1) |
| 16 | eastern chimpanzee associated porprismacovirus 4 | 23.3 (14.3, 35.6) | 17.7 (5.4, 41.8) | 22.7 (9.7, 43.9) | 28.6 (13.6, 50.2) | 20.0 (9.1, 37.7) | 26.7 (14.0, 44.7) |
| 17 | eastern chimpanzee associated porprismacovirus 6 | 18.3 (10.4, 30.1) | 17.7 (5.4, 41.8) | 18.2 (6.7, 39.1) | 19.1 (7.1, 40.6) | 23.3 (11.5, 41.2) | 13.3 (4.7, 30.3) |
| 18 | eastern chimpanzee associated porprismacovirus 7 | 6.7 (2.2, 16.4) | 11.8 (2.0, 35.6) | 9.1 (1.3, 29.0) | 0.0 (0.0, 18.2) | 10.0 (2.7, 26.4) | 3.3 (<0.1, 18.1) |
| 19 | eastern chimpanzee associated porprismacovirus 8 | 18.3 (10.4, 30.1) | 11.8 (2.0, 35.6) | 13.6 (3.9, 34.2) | 28.6 (13.6, 50.2) | 13.3 (4.7, 30.3) | 23.3 (11.5, 41.2) |
| 20 | eastern chimpanzee associated porprismacovirus 9 | 33.3 (22.7, 46.0) | 29.4 (13.0, 53.4) | 36.4 (19.6, 57.1) | 33.3 (17.1, 54.8) | 33.3 (19.1, 51.3) | 33.3 (19.1, 51.3) |
| 21 | eastern chimpanzee associated porprismacovirus 10 | 8.3 (3.2, 18.5) | 0.0 (0.0, 21.6) | 9.1 (1.3, 29.0) | 14.3 (4.1, 35.5) | 10.0 (2.7, 26.4) | 6.7 (0.8, 22.4) |
| 22 | eastern chimpanzee associated porprismacovirus 11 | 11.7 (5.5, 22.5) | 5.9 (<0.1, 28.9) | 18.2 (6.7, 39.1) | 9.5 (1.5, 30.1) | 13.3 (4.7, 30.3) | 10.0 (2.7, 26.4) |
| 23 | eastern chimpanzee associated porprismacovirus 12 | 8.3 (3.2, 18.5) | 11.8 (2.0, 35.6) | 4.6 (<0.1, 23.5) | 9.5 (1.5, 30.1) | 13.3 (4.7, 30.3) | 3.3 (<0.1, 18.1) |
| 24 | unclassified circular ssDNA virus 3 | 1.7 (<0.1, 9.7) | 0.0 (0.0, 21.6) | 4.6 (<0.1, 23.5) | 0.0 (0.0, 18.2) | 0.0 (0.0, 13.5) | 3.3 (<0.1, 18.1) |
| 25 | unclassified circular ssDNA virus 4 | 11.7 (5.5, 22.5) | 11.8 (2.0, 35.6) | 4.6 (<0.1, 23.5) | 19.1 (7.1, 40.6) | 23.3 (11.5, 41.2) | 0.0 (0.0, 13.5) |
| 26 | unclassified circular ssDNA virus 5 | 13.3 (6.7, 24.4) | 0.0 (0.0, 21.6) | 13.6 (3.9, 34.2) | 23.8 (10.2, 45.5) | 26.7 (14.0, 44.7) | 0.0 (0.0, 13.5) |
| 27 | unidentified circular ssDNA virus 6 | 3.3 (0.3, 12.0) | 0.0 (0.0, 21.6) | 0.0 (0.0, 17.6) | 9.5 (1.5, 30.1) | 6.7 (0.8, 22.4) | 0.0 (0.0, 13.5) |

**Table S2**. Variation in viral richness analyzed by (a) linear mixed model and (b) robust linear mixed model. The marginal and conditional R^2^ for the linear mixed model were 0.21 and 0.63, respectively. The corresponding reference categories for “Reproductive Status” and “Community” were “Cycling” and “Kanyawara”. Bold font indicates predictors for which p < 0.05.

| (a) Linear Mixed Model | | | | | | |  |
| --- | --- | --- | --- | --- | --- | --- | --- |
| Predictor |  | β | SE | 95% CI | DF | t | p |
| Intercept |  | 3.72 | 0.853 | [2.04, 5.34] |  |  |  |
| Reproductive Status: | Pregnant | 0.041 | 0.658 | [-1.24, 1.44] | 21.5 | 0.062 | 0.951 |
|  | **Lactating** | **1.96** | **0.720** | **[0.546, 3.38]** | **20.3** | **2.72** | **0.013** |
| Age |  | 0.030 | 0.320 | [-0.682, 0.687] | 20.4 | 0.094 | 0.926 |
| **Community (Ngogo)** | | **-1.62** | **0.709** | **[-2.98, -0.163]** | **22.4** | **-2.29** | **0.032** |
|  |  |  |  |  |  |  |  |
| (b) Robust Linear Mixed Model | | | | | | |  |
| Predictor |  | β | SE | - | - | t | p |
| Intercept |  | 3.56 | 0.853 | - | - |  |  |
| Reproductive Status: | Pregnant | 0.340 | 0.782 | - | - | 0.435 | 0.666 |
|  | **Lactating** | **1.80** | **0.858** | **-** | **-** | **2.10** | **0.043** |
| Age |  | 0.281 | 0.317 | - | - | 0.888 | 0.387 |
| **Community** |  | **-1.61** | **0.697** | **-** | **-** | **-2.31** | **0.034** |

**Table S3**. Variation in total viral load analyzed by (a) linear mixed model and (b) robust linear mixed model. The marginal and conditional R^2^ for the linear mixed model were 0.23 and 0.60, respectively. The reference categories for “Reproductive Status” and “Community” were “Cycling” and “Kanyawara”, respectively. Bold font denotes predictors for which p < 0.05.

| (a) Linear Mixed Model | | | | | | |  |
| --- | --- | --- | --- | --- | --- | --- | --- |
| Predictor |  | β | SE | 95% CI | DF | t | p |
| Intercept |  | 4.39 | 0.353 | [3.76, 5.08] |  |  |  |
| Reproductive Status: | Pregnant | 0.000 | 0.366 | [-0.685, 0.866] | 15.9 | 0.001 | 0.999 |
|  | Lactating | 0.588 | 0.346 | [-0.079, 1.33] | 15.4 | 1.70 | 0.109 |
| Age |  | 0.047 | 0.126 | [-0.188, 0.289] | 12.5 | 0.371 | 0.717 |
| **Community** |  | **-0.934** | **0.281** | **[-1.51, -0.409]** | **11.4** | **-3.33** | **0.006** |
|  |  |  |  |  |  |  |  |
| (b) Robust Linear Mixed Model | | | | | | |  |
| Predictor |  | β | SE | - | - | t | p |
| Intercept |  | 4.50 | 0.323 |  |  |  |  |
| Reproductive Status: | Pregnant | 0.080 | 0.371 |  |  | 0.215 | 0.831 |
|  | Lactating | 0.599 | 0.375 |  |  | 1.60 | 0.118 |
| Age |  | 0.074 | 0.146 |  |  | 0.505 | 0.623 |
| **Community** |  | **-1.08** | **0.293** |  |  | **-3.68** | **0.007** |

**Table S4.** Variable importance derived from random forests assessing (1) richness in lactating females by viral prevalence, (2) study community by viral prevalence, and (3) study community by viral load (vRPM/kb), respectively. Viruses are organized by importance values in category “Lactation: Prevalence” from largest to smallest.

| **Virus** | **Lactation: Prevalence^1^** | **Community: Prevalence^2^** | **Community: Viral Load^3^** |
| --- | --- | --- | --- |
| unclassified circular ssDNA virus 5 | 9.541 | 1.178 | 1.200 |
| eastern chimpanzee associated porprismacovirus 2 | 9.236 | 0.413 | 0.634 |
| eastern chimpanzee associated porprismacovirus 6 | 7.387 | 0.428 | 0.516 |
| unclassified circular ssDNA virus 4 | 7.167 | 0.998 | 0.975 |
| eastern chimpanzee associated porprismacovirus 8 | 4.671 | 0.503 | 0.484 |
| chimpanzee picobirna-like virus | 4.021 | 0.190 | 0.170 |
| eastern chimpanzee associated porprismacovirus 4 | 3.756 | 0.454 | 0.470 |
| chimpanzee enterovirus D | 3.571 | 0.399 | 0.648 |
| eastern chimpanzee associated porprismacovirus 1 | 2.569 | 0.331 | 0.585 |
| eastern chimpanzee associated porprismacovirus 12 | 2.380 | 0.325 | 0.515 |
| chimpanzee circovirus 2 | 2.343 | 1.078 | 1.796 |
| eastern chimpanzee associated porprismacovirus 9 | 1.675 | 0.320 | 0.915 |
| chimpanzee adenovirus | 1.530 | 0.334 | 0.579 |
| unidentified circular ssDNA virus 6 | 1.331 | 0.138 | 0.126 |
| eastern chimpanzee associated porprismacovirus 11 | 1.097 | 0.253 | 0.395 |
| chimpanzee genomovirus | 1.094 | 0.525 | 0.511 |
| eastern chimpanzee associated porprismacovirus 3 | 0.925 | 0.062 | 0.046 |
| chimpanzee circovirus 1 | 0.616 | 0.356 | 1.136 |
| eastern chimpanzee associated porprismacovirus 10 | 0.547 | 0.404 | 0.359 |
| chimpanzee salivirus | 0.405 | 0.296 | 0.615 |
| chimpanzee bufavirus (protoparvovirus) | 0.225 | 0.895 | 0.939 |
| chimpanzee kobuvirus | 0.078 | 0.108 | 0.129 |
| chimpanzee enterovirus A | 0.000 | 0.243 | 0.184 |
| chisavirus (chimpanzee stool-associated RNA virus) | 0.000 | 0.177 | 0.110 |
| eastern chimpanzee associated porprismacovirus 7 | 0.000 | 0.269 | 0.240 |
| chimpanzee astrovirus | - | 0.052 | 0.037 |
| unclassified circular ssDNA virus 3 | - | 0.051 | 0.040 |

**References**

Abascal, F., Zardoya, R. & Telford, M.J. (2010) TranslatorX: multiple alignment of nucleotide sequences guided by amino acid translations. *Nucleic Acids Research,* **38,** W7-W13. https://doi.org/10.1093/nar/gkq291

Altschul, S.F., Gish, W., Miller, W., Myers, E.W. & Lipman, D.J. (1990) Basic local alignment search tool. *Journal of Molecular Biology,* **215,** 403-410. https://doi.org/10.1016/S0022-2836(05)80360-2

Castresana, J. (2000) Selection of conserved blocks from multiple alignments for their use in phylogenetic analysis. *Molecular Biology and Evolution,* **17,** 540-552. https://doi.org/10.1093/oxfordjournals.molbev.a026334

Geniole, S.N., Proietti, V., Bird, B.M., Ortiz, T.L., Bonin, P.L., Goldfarb, B., Watson, N.V. & Carré, J.M. (2019) Testosterone reduces the threat premium in competitive resource division. *Proceedings of the Royal Society B: Biological Sciences,* **286,** 20190720. https://doi.org/10.1098/rspb.2019.0720

Gish, W. & States, D.J. (1993) Identification of protein coding regions by database similarity search. *Nature Genetics,* **3,** 266-272. https://doi.org/10.1038/ng0393-266

Goldberg, T.L., Bennett, A.J., Kityo, R., Kuhn, J.H. & Chapman, C.A. (2017) Kanyawara virus: A novel rhabdovirus infecting newly discovered nycteribiid bat flies infesting previously unknown pteropodid bats in Uganda. *Scientific Reports,* **7,** 5287-5287. https://doi.org/10.1038/s41598-017-05236-w

Goldberg, T.L., Clyde, V.L., Gendron-Fitzpatrick, A., Sibley, S.D. & Wallace, R. (2018) Severe neurologic disease and chick mortality in crested screamers (*Chauna torquata*) infected with a novel *Gyrovirus*. *Virology,* **520,** 111-115. https://doi.org/10.1016/j.virol.2018.05.014

Goldberg, T.L., Sibley, S.D., Pinkerton, M.E., Dunn, C.D., Long, L.J., White, L.C. & Strom, S.M. (2019) Multidecade mortality and a homolog of Hepatitis C virus in bald eagles (*Haliaeetus leucocephalus*), the national bird of the USA. *Scientific Reports,* **9,** 14953. https://doi.org/10.1038/s41598-019-50580-8

Koller, M. (2016) robustlmm: An R package for robust estimation of linear mixed-effects models. *Journal of Statistical Software,* **75,** 1-24. https://doi.org/10.18637/jss.v075.i06

Lefort, V., Longueville, J.-E. & Gascuel, O. (2017) SMS: Smart model selection in PhyML. *Molecular Biology and Evolution,* **34,** 2422-2424. https://doi.org/10.1093/molbev/msx149

Liaw, A. & Wiener, M. (2002) Classification and regression by randomForest. *R News,* **2,** 18-22.

Luke, S.G. (2017) Evaluating significance in linear mixed-effects models in R. *Behavior Research Methods,* **49,** 1494-1502. https://doi.org/10.3758/s13428-016-0809-y

Negrey, J.D., Emery Thompson, M., Langergraber, K.E., Machanda, Z.P., Mitani, J.C., Muller, M.N., Otali, E., Owens, L.A., Wrangham, R.W. & Goldberg, T.L. (2020) Demography, life history trade-offs, and the gastrointestinal virome of wild chimpanzees. *Philosophical Transactions of the Royal Society B,* **375,** 20190613. https://doi.org/10.1098/rstb.2019.0613

Negrey, J.D., Mitani, J.C., Wrangham, R.W., Otali, E., Reddy, R.B., Pappas, T.E., Grindle, K.A., Gern, J.E., Machanda, Z.P., Muller, M.N., Langergraber, K.E., Emery Thompson, M. & Goldberg, T.L. (2022) Viruses associated with ill health in wild chimpanzees. *American Journal of Primatology,* **84,** e23358. https://doi.org/10.1002/ajp.23358

Rambaut, A. (2018) FigTree, version 1.4.4.

Segal, M. & Xiao, Y. (2011) Multivariate random forests. *WIREs Data Mining and Knowledge Discovery,* **1,** 80-87. https://doi.org/10.1002/widm.12

Shapiro, S.S. & Wilk, M.B. (1965) An analysis of variance test for normality (complete samples). *Biometrika,* **52,** 591-611. https://doi.org/10.1093/biomet/52.3-4.591

Sibley, S.D., Finley, M.A., Baker, B.B., Puzach, C., Armién, A.G., Giehtbrock, D. & Goldberg, T.L. (2016) Novel reovirus associated with epidemic mortality in wild largemouth bass (*Micropterus salmoides*). *Journal of General Virology,* **97,** 2482-2487. https://doi.org/10.1099/jgv.0.000568

Toohey-Kurth, K., Sibley, S.D. & Goldberg, T.L. (2017) Metagenomic assessment of adventitious viruses in commercial bovine sera. *Biologicals,* **47,** 64-68. https://doi.org/10.1016/j.biologicals.2016.10.009
